# Paraneoplastic dermatomyositis and Hodgkin’s lymphoma in a 14-year-old girl: a case report and literature review

**DOI:** 10.3389/fonc.2024.1416083

**Published:** 2024-08-07

**Authors:** Yanyan Ling, Huaiqiang Hu, Xiangyan Xu, Jianli Feng, Mingzhe Li, Huan Li, Ming Cheng, Xiaoling Wang

**Affiliations:** ^1^ Department of Neurology, Shandong Second Provincial General Hospital, Jinan, China; ^2^ Department of Neurology, The 960th Hospital of Joint Logistics Force, PLA, Jinan, China; ^3^ Department of Traumatic Orthopedics, Shandong Second Provincial General Hospital, Jinan, China; ^4^ Department of Emergency, Shandong Second Provincial General Hospital, Jinan, China; ^5^ Department of Neurology, The 970th Hospital of Joint Logistics Force, PLA, Yantai, China

**Keywords:** juvenile dermatomyositis, Hodgkin’s lymphoma, paraneoplastic syndrome, malignancy, nodular sclerosing Hodgkin’s lymphoma

## Abstract

**Background:**

Juvenile dermatomyositis (JDM) is a rare autoimmune myopathy whose main clinical manifestations include a characteristic rash, symmetrical proximal muscle weakness, and elevated muscle enzymes. While approximately one-third of adult patients with dermatomyositis (DM) develop malignancies, typically within a year of diagnosis, this phenomenon is not commonly observed in patients with JDM. In this study, we present a rare case of both JDM and Hodgkin’s lymphoma (HL) diagnosed in an adolescent female patient.

**Case description:**

A 14-year-old girl with proximal muscle weakness and myalgia for 8 weeks was admitted to the hospital and ultimately received a diagnosis of DM. A thorough physical examination revealed enlarged lymph nodes on both sides of the cervical, and a lymph node biopsy was performed to diagnose HL. After she underwent radiotherapy and chemotherapy, her symptoms of both HL and DM were alleviated.

**Conclusion:**

The phenomenon of JDM as a paraneoplastic syndrome associated with HL is very rare. Thus, routine cancer screening for DM in adolescents is currently not recommended. The diagnosis of JDM requires a detailed physical examination, and further tumor screening is necessary for patients with unusual physical findings, such as atypical rashes, enlarged lymph nodes, and enlargement of the spleen and/or liver. Even if no malignancy is detected when JDM is diagnosed, long-term follow-up is necessary.

## Introduction

Juvenile dermatomyositis (JDM)—an immune-mediated multisystem disease characterized by acute and chronic non-suppurative inflammation affecting the skin, striated muscles, lungs, and gastrointestinal tract (1)—is the most common idiopathic inflammatory myopathy (IIM) in children. Typical clinical manifestations include a lilac rash on the eyelids, Gottron’s sign, and symmetrical proximal muscle weakness of the limbs. Additionally, significant elevation of muscle enzymes, such as creatine kinase (CK), is observed (2, 3). Epidemiological data indicate an annual incidence of 2 to 4 cases per million children (4), with the onset typically peaking between the ages of 5 and 10 years and girls being more commonly affected than boys, with an estimated ratio of 2-5:1 (5). In adults, the association between dermatomyositis (DM) and malignancy is well known, with a reported prevalence of 7 to 60%, especially in those demonstrating disease onset after the age of 50 (6, 7). Therefore, extensive screening for occult cancers is common for adults with DM. In contrast, an association between DM and malignancy has rarely been noted in children. Here, we present a case of JDM in a patient with Hodgkin’s lymphoma (HL) as a paraneoplastic manifestation. Additionally, we conducted a PubMed search and retrieved reports of four cases of HL combined with JDM. Our case report clarifies whether routine evaluation of malignant tumors is recommended for patients with JDM and the circumstances under which further screening for malignant tumors is required.

## Case presentation

A 14-year-old girl with obesity experiencing symmetrical proximal limb muscle weakness and myalgia for 8 weeks was admitted to the 960th Hospital of Joint Logistics Force, PLA. Cutaneous manifestations included a lilac rash around both eyes and a flaky V-shaped rash on the chest. The patient did not have speaking or breathing difficulties, a family history of autoimmune disease or malignancy, or a history of fever before the onset of muscle pain. Her development was otherwise normal.

**Figure 1:**
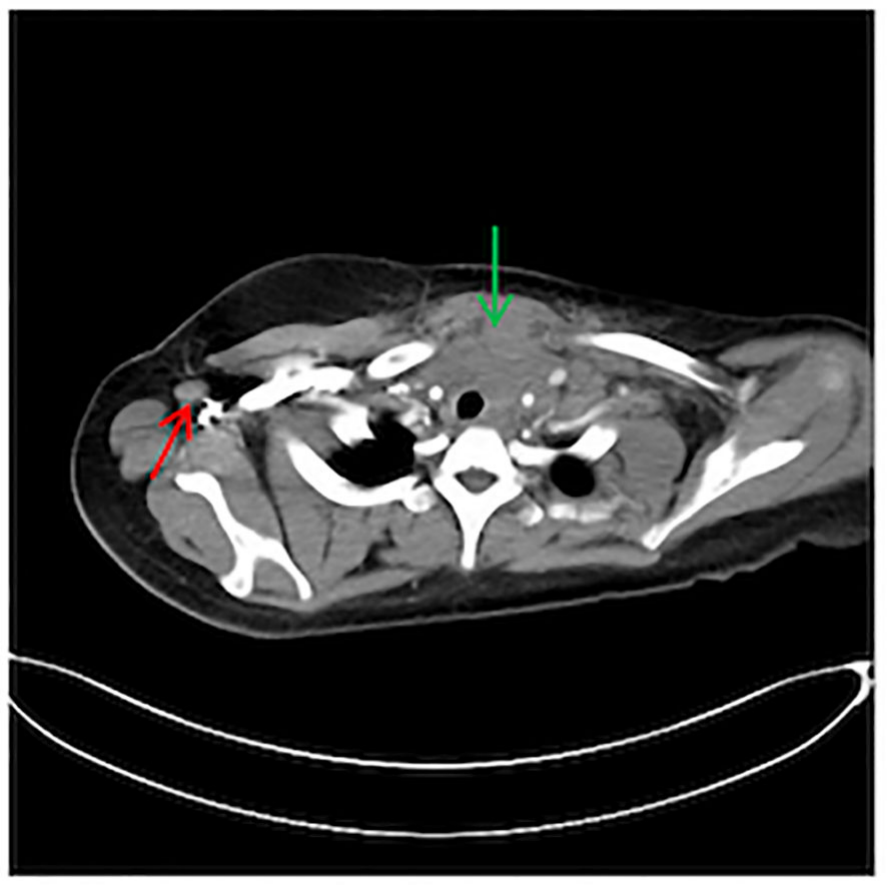
Chest computed tomography (CT) showing an anterior mediastinal mass (Green) and right axillary lymph node enlargement (Red).

**Figure 2:**
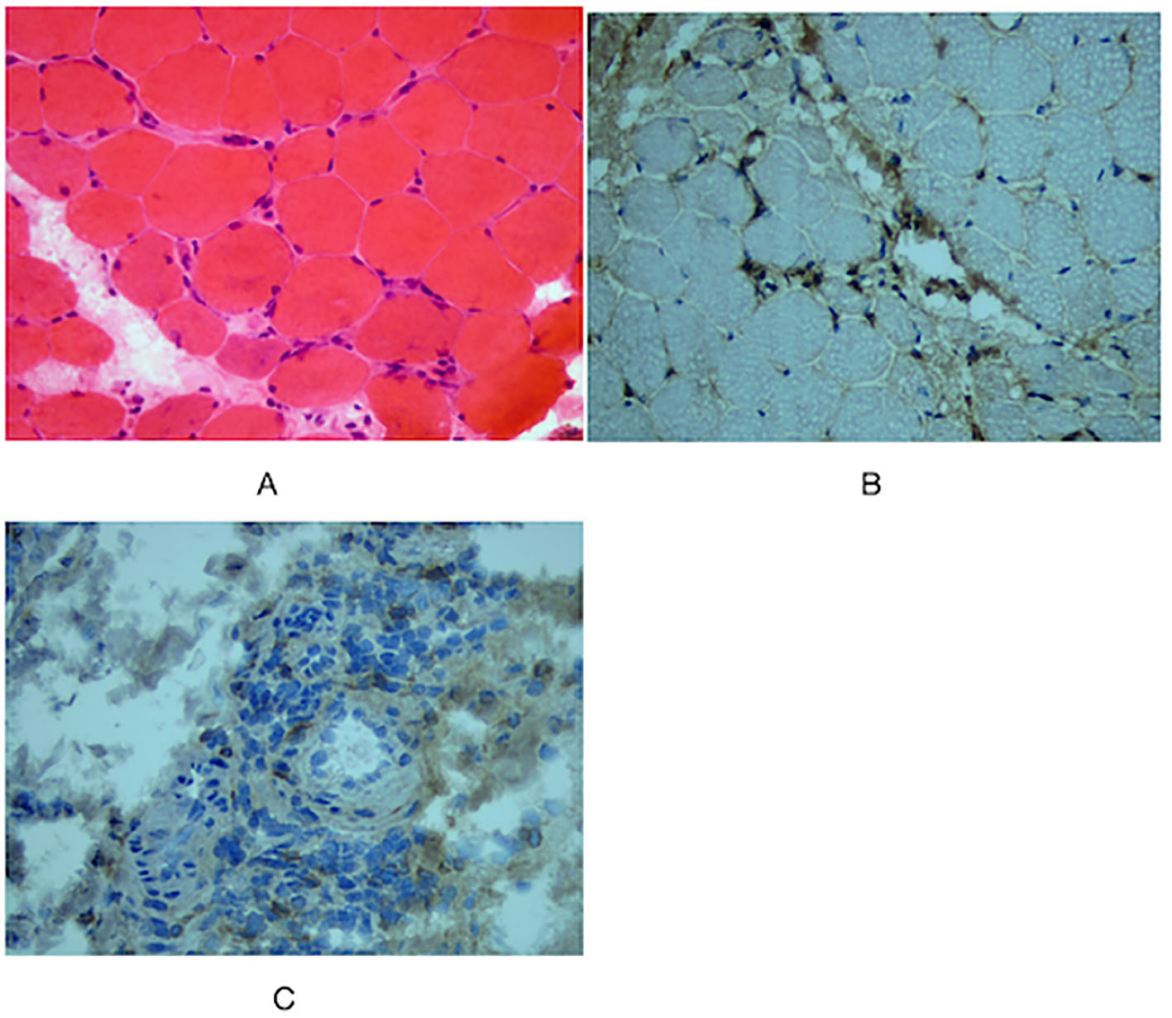
Muscle biopsy of left musculus biceps brachii. **(A)** Hematoxylin and Eosin (HE) staining showed perifascicular atrophy of muscle fibers, and perivascular inflammatory infiltrates (x40). No regenerating muscle fibers are observed, and there is no apparent nuclear migration of muscle fibers. Immunohistochemical staining showed **(B)** CD4+, **(C)** CD68+ (×40).

**Figure 3:**
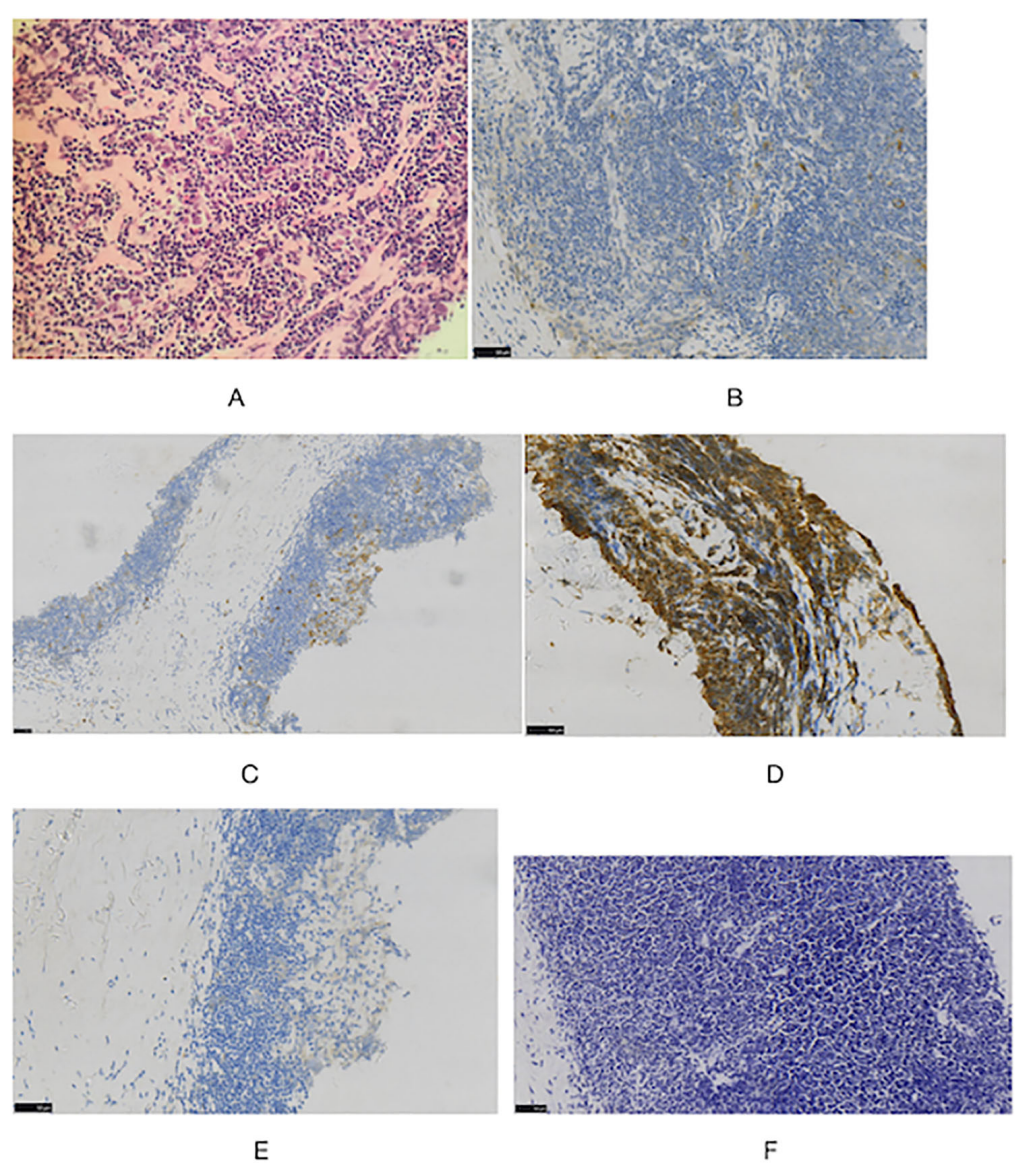
Histologic images in cervical lymph node **(A)** staining showed typical Hodgkin Reed-Sternberg (H-RS) cells in a variable inflammatory background. (HE, x40). Immunohistochemical staining also showed **(B)** CD30(+), **(C)** CD15(+), **(D)** CD20(+), **(E)** ALK(-) **(F)** EBER(-) (×40).

**Figure 4:**
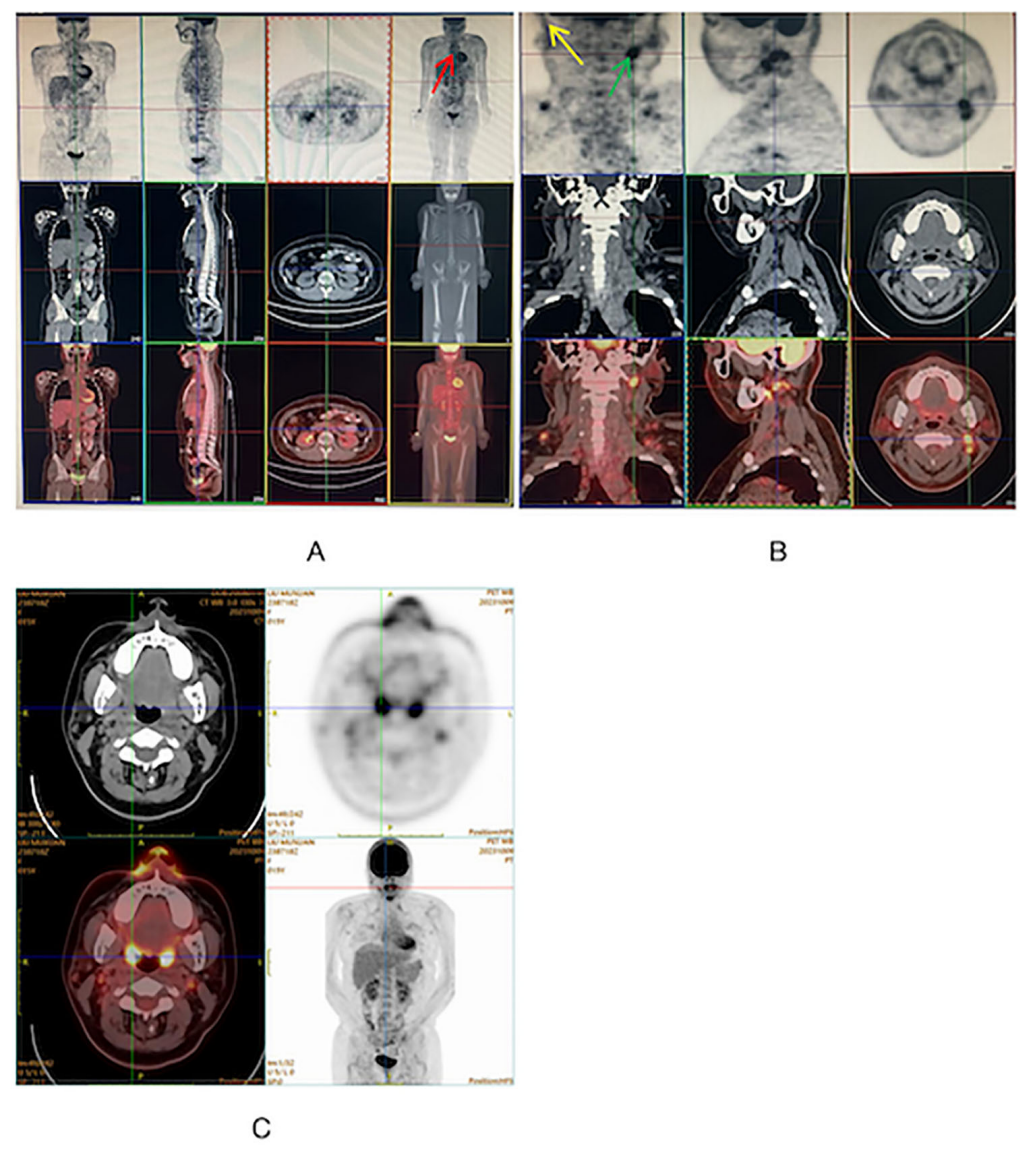
**(A, B)** FDG-PET on admission showing diffuse uptake in mediastinum supradiaphragmatic lymph nodes (Red), left supraclavicular lymph node (Yellow) and right cervical lymph nodes (Green). **(C)** FDG-PET at 11 months after diagnosis showing disappearance of abnormal signals.

During the physical examination, the patient was found to experience pain in the extremities upon pressing. She also exhibited generalized edema, including periorbital swelling and bilateral non-pitting edema of the upper and lower limbs. Bilateral cervical lymph node enlargement was detected on palpation; however, there was no evidence of organomegaly. The Manual Muscle Testing (MMT-8) score was 76, with the following specific muscle group scores: 8 for elbow flexors, 8 for hip flexors, 10 for shoulder abductors, 10 for wrist extensors, 10 for ankle dorsiflexors, 10 for neck flexors, 10 for hip abductors, and 10 for hip extensors (8). Electromyography (EMG) findings were consistent with the diagnosis of inflammatory muscle disease. To further evaluate the bilateral cervical lymphadenopathy noted during the physical examination, a chest computed tomography (CT) scan was performed. CT revealed an anterior mediastinal mass, bilateral subclavian lymph node enlargement, and bilateral axillary lymph node enlargement (**Figure 1**). Biopsy of the left brachii muscle showed evidence consistent with the presence of DM (**Figure 2**), and cervical lymph node biopsy indicated nodular sclerosing HL (nsHL) (**Figure 3**). F-fluorodeoxyglucose positron emission tomography (FDG-PET) revealed mild diffuse increases in subcutaneous FDG metabolism in the extremities, along with anterior diaphragmatic lymphadenopathy and involvement of the sternal stalk (**Figure 4**). Bone marrow aspiration results showed no abnormalities.

Laboratory investigations revealed an elevated C-reactive protein (CRP) level of 12.40 mg/L (normal range: 0–5 mg/L). Tests for anti-nuclear antibodies (ANAs), anti-double-stranded DNA (anti-dsDNA), and anti-extractable nuclear antigen antibodies (including anti-Jo-1, anti-PM-Scl 70, anti-PM-Scl 100, anti-Ku, anti-Ro-52, anti-SSA, anti-SSB, and anti-Sm) were negative, as were serology tests for myositis-specific antibodies (MSAs) (anti-Mi-2, -Jo1, -EJ, -PL-12, -PL-7, -SAE 1/2, -MDA5, -SRP, -HMGCR, -TIF-1γ, -SSA/Ro52, and -NXP-2). Line-blot for MSAs detection in JDM serum. Serum samples were tested by the commercial line blot Simcere Diagnostics autoimmune myositis 12 Ag (MYO12D-12) following the manufacturers’ instructions. Test strips were scanned and evaluated for band intensity using the LineScan software (Dr DOT4). Following the manufacturer’s recommendations, classified as negative (-) or positive (+). Negative: <5 AU (arbitrary units); gray zone: 5-10 AU (retest after 8-12 weeks); positive: >10 AU. CK, lactate dehydrogenase (LDH), alanine transaminase (ALT), and aspartate transaminase (AST) levels; white blood cell (WBC), red blood cell (RBC), and platelet (PLT) counts; and erythrocyte sedimentation rate (ESR) were within the normal ranges.

Ultimately, stage IV A nsHL concurrent with JDM was diagnosed. Chemotherapy was initiated according to the protocol for stage IV A, which comprised a regimen of adriamycin, bleomycin, vinblastine, and dacarbazine (ABVD). Prednisolone (2 mg/kg/day, orally) was administered for 2 months to treat JDM. The patient underwent six courses of chemotherapy, along with 15 sessions of local radiation therapy, to treat HL. Eleven months after the diagnosis, HL and JDM were completely in remission (**Figure 4**).

## Discussion

JDM was diagnosed in our patient who met four of the five Bohan and Peter criteria: typical skin changes, proximal muscle weakness, myopathic changes detected by EMG, and abnormal muscle biopsy findings (9). She also met the diagnostic criteria of the European League Against Rheumatism/American College of Rheumatology (EULAR/ACR) with a score of 10.7, thus falling in the “definite IIM” category (aggregate score ≥ 8.7) (2). However, only a few cases of patients with JDM either having DM as a paraneoplastic manifestation or developing a lymphoma after diagnosis of JDM have been reported. While the link between DM and lymphoma is well documented in adults, it has rarely been observed in adolescent patients with JDM.

We conducted a search on PubMed for case reports and review literature using the following terms: juvenile dermatomyositis, myositis, malignancies, cancer, tumor, children, adolescents, and pediatrics, either individually or in combination. This search yielded the case reports of four pediatric patients with JDM and lymphoma (10–13). **Table 1** shows the characteristics of these four cases and ours (patient 5). Three of these patients were girls, and the remaining two were boys, with an average age of 13.8 years. JDM and HL were diagnosed in three patients (patients 1, 4, and 5) simultaneously, all of whom demonstrated unusual physical findings at the time of JDM diagnosis, including enlarged lymph nodes. The other two patients (patients 2 and 3) exhibited no atypical rash, lymphadenopathy, or splenomegaly and/or hepatomegaly as unusual physical findings at the time of JDM diagnosis. The patient 2 was found to have a right supraclavicular mass 13 months after being diagnosed with JDM, and was diagnosed HL IIA. The patient 3 was found to have a painless left supraclavicular lymph node 5 months after being diagnosed with JDM. The biopsy result of this supraclavicular lymph node was consistent with HL.

**Table 1:** Presentation of lymphoma and JDM in pediatric patients.

Patient	Age/years	Sex	JDM:Rx	Unusual Physical Findings	Lymphoma Stage	Interval/month	Lymphoma:Rx	Outcome/month	References
1	12y	Female	Prednisone	Lymphadenopathy	HL IIB	0 (concurrent)	COPP	Remission (30m)	10
2	18y	Male	Prednisone, HCQ, MTX	None	HL IIA	13m	RT	Remission (6m)	11
3	14y	Male	Prednisone	None	nsHL 1A	5m	RT	Remission (18m)	12
4	11y	Female	Prednisone,IVIG	Lymphadenopathy	HL IIE	0 (concurrent)	OPPA,COPP, RT	Remission (24m)	13
5	14y	Female	Prednisone	Lymphadenopathy	nsHL IVA	0 (concurrent)	ABVD,RT	Remission (11m)	

JDM, Juvenile dermatomyositis; HL, Hodgkin’s lymphoma; nsHL, nodular sclerosing Hodgkin’s lymphoma; MTX, Methotrexate; RT, Radiation therapy; HCQ, hydroxychloroquine; IVIG, Intravenous immunoglobulin; OPPA, Vincristine/Procarbazine/Predonisone/Doxorubicin; COPP, Cyclophosphamide/Vincristine/Predonisone/Procarbazine; ABVD, Adriamycin/Bleomycin;/Vincristine;/Azenimide.

The pathogenesis of JDM and lymphoma has not been fully elucidated, and multiple mechanisms may be attributed to the link between the two. First, long-term medication-induced immunosuppression (14) has been suggested as a contributing factor. Stübgen described five patients whose lymphoma regressed only upon withdrawal of such drugs and when combined with locoregional radiation, supporting the notion of treatment-associated lymphoma (15). Second, high inflammatory activity in patients with autoimmune diseases is considered a major risk factor for developing lymphoma (16). Additionally, the occurrence of JDM as a paraneoplastic phenomenon associated with lymphoma has rarely been reported (17). Evidence indicating a potential association between JDM and malignancy is limited, which is in contrast with that demonstrated in the adult population (14). A majority of the studies regarding the association between JDM and malignant tumors consist of isolated case reports. In a comprehensive review of the literature spanning 45 years, 12 cases establishing an association between JDM/polymyositis (PM) and malignancy were identified. It showed that the occurrence of a paraneoplastic syndrome can be concluded if a malignant tumor occurs within 12 months of diagnosing DM and/or successful tumor treatment can improve the symptoms of DM (18). Among the five cases described in **Table 1**, three (patients 1, 4, and 5) support the hypothesis that JDM is a paraneoplastic phenomenon (13). This is evidenced by the simultaneous diagnosis of JDM and HL, with the lymphoma already at stage II or IV when diagnosed. Subsequent radiotherapy and chemotherapy treatment alleviated symptoms of DM, such as muscle weakness, pain, and rash. Patient 3 was diagnosed with HL within 12 months of being diagnosed with JDM, suggesting a paraneoplastic phenomenon. However, patient 2 had a malignancy that was less clearly temporally related to the JDM diagnosis than it was in the other three patients (12, 18).

The etiology of JDM as a paraneoplastic phenomenon of lymphoma remains unknown; however, some mechanisms have been proposed. First, environmental factors can trigger both cancer and myositis in genetically susceptible hosts (15). Second, malignant cells in HL secrete cytokines excessively. Activated cytokines enable T cell-mediated myocyte toxicity or complement-mediated microangiopathy, and the tumor products cause muscle and skin inflammation (19). Third, cross-reactions between tumors and muscle or skin antigens can cause dysregulation of the immune system (20–22).

Previous studies have shown that anti-TIF-1γ autoantibodies are one of the most common MSAs in adult DM and JDM patients (23, 24). DM patients, adult or adolescent, with positive anti-TIF-1γ autoantibodies often present with extensive and severe skin lesions, including photosensitive rashes, skin ulcers, and muscle atrophy (25). Importantly, in adults with DM, anti-TIF-1γ autoantibodies are associated with an increased risk of cancer (23). According to previous studies, the positivity rate of anti-TIF-1γ antibodies in patients with DM-associated tumors is over 50%, confirming their reliability as a predictor of malignancy in DM (26). However, no association between anti-TIF-1γ autoantibodies and malignant tumors has been found in adolescent patients with JDM (27). Research indicates that patients, whether adults or adolescents, who test positive for anti-TIF-1γ may also exhibit other autoantibodies, such as anti-Sp4, anti-CCAR1, and anti-TBL1XR1 autoantibodies (28, 29). Patients with JDM who present with anti-TIF-1γ antibodies and associated autoantibodies exhibit less muscle weakness, less frequent skin ulcers, and lower anti-TIF-1γ autoantibody titers (29) perhaps because, in this subgroup of patients, the titer of anti-TIF-1γ autoantibodies appears to decrease due to other autoantibodies targeting antigens that form complexes with TIF-1γ (28). Therefore, the potential myotoxicity effect of anti-TIF1-γ autoantibodies may be weakened. Results reported in different studies show that autoantibodies against Sp4 and CCAR1 are associated with a reduced risk of cancer in adults with anti-TIF-1γ -positive DM. The specific mechanism by which the presence of associated antibodies in such patients reduces tumor risk is unclear, but it may be that they are eradicated more efficiently in those who mount an immune response against both TIF-1γ and the associated autoantigens (28).

JDM is a rare paraneoplastic syndrome, and large-scale data regarding the relationship between MSAs and malignancy are currently lacking. However, unusual physical findings during diagnosis of JDM, such as atypical rash, organomegaly, or lymphadenopathy, may necessitate a thorough evaluation to exclude malignancy. We observed unusual features, including lymphadenopathy, in our patient. Patients 2 and 3 had no unusual physical findings at the time of diagnosis of JDM, while HL was detected 13 and 5 months later, respectively (11). Hence, even if a malignancy is not detected when JDM is diagnosed, follow-up is necessary. Early identification is critical in such patients because steroid treatment can partially inhibit tumor growth, potentially delaying the establishment of a correct diagnosis, which could negatively impact the patient’s prognosis.

## Conclusion

Here, we described a rare case of JDM as a paraneoplastic syndrome of HL, but only a few cases of JDM as a paraneoplastic manifestation have been reported, and thus, routine screening for lymphoma in patients with JDM does not seem to be warranted. Unusual physical findings during the diagnosis of JDM may indicate the presence of a malignant tumor, necessitating further evaluation to screen for malignancy. Even if no malignant tumor is found at the time of JDM diagnosis, follow-up is necessary. Regular follow-up of JDM patients should not solely focus on the characteristics of myositis but should also encompass a systematic and comprehensive physical examination. Comprehensive malignancy screening is required if any unusual physical findings are observed during follow-up. Further study of paraneoplastic syndromes in children, focusing on pathophysiology and treatment strategies, is required.

## Data availability statement

The original contributions presented in the study are included in the article supplementary material. Further inquiries can be directed to the corresponding author.

## Ethics statement

The studies involving humans were approved by The 960 Hospital of the PLA Joint Logistic Support Force. The studies were conducted in accordance with the local legislation and institutional requirements. Written informed consent for participation in this study was provided by the participants’ legal guardians/next of kin. Written informed consent was obtained from the minor(s)’ legal guardian/next of kin for the publication of any potentially identifiable images or data included in this article.

## Author contributions

YL: Formal Analysis, Writing – original draft. HH: Software, Writing – review & editing. XX: Methodology, Supervision, Writing – review & editing. JF: Investigation, Supervision, Writing – review & editing. ML: Formal Analysis, Methodology, Writing – review & editing. HL: Formal Analysis, Methodology, Writing – review & editing. MC: Formal Analysis, Investigation, Supervision, Writing – review & editing. XW: Formal Analysis, Investigation, Methodology, Supervision, Writing – review & editing.
